# Atraumatic Tension Pneumocephalus in a Shunted Patient: A Case of Rapid Neurological Decline

**DOI:** 10.7759/cureus.92633

**Published:** 2025-09-18

**Authors:** Lily D Rundquist, Jomaris O Gomez-Rosado, Christopher Nunez, Aleksandr Dubrovskiy

**Affiliations:** 1 Medicine, Dr. Kiran C. Patel College of Osteopathic Medicine, Nova Southeastern University, Fort Lauderdale, USA; 2 Internal Medicine, Cleveland Clinic Weston Hospital, Weston, USA; 3 Emergency Medicine, Broward North Hospital, Pompano Beach, USA

**Keywords:** cerebral spinal fluid (csf), pneumocephalus, subdural hematoma, tension pneumocephalus, vp shunt complication

## Abstract

Pneumocephalus is a rare but potentially life-threatening condition caused by the presence of air within the intracranial cavity. It commonly arises from trauma, neurosurgical procedures, infections, malignancies, or spontaneous causes. In patients with ventriculoperitoneal (VP) shunts, pneumocephalus may result from complications such as altered cerebrospinal fluid dynamics or skull base defects leading to a vacuum-like effect. We report a case of an 83-year-old male with a history of VP shunt placement for normal pressure hydrocephalus who presented with acute neurological deterioration, including aphasia and quadriparesis. Imaging revealed severe frontal pneumocephalus with mass effect and midline shift. The patient underwent emergent burr hole evacuation and VP shunt removal. Despite surgical intervention, he developed a new sizable subdural hematoma, leading to further deterioration. Following a prolonged critical course and poor neurological recovery, the patient was transitioned to hospice care. This case highlights the potential for severe tension pneumocephalus in patients with VP shunts, emphasizing the need for early recognition and prompt neurosurgical management. Given the high morbidity associated with tension pneumocephalus, clinicians should maintain vigilance for this rare but serious complication.

## Introduction

Pneumocephalus, the presence of air within the intracranial cavity, is a rare but potentially life-threatening condition that can lead to significant neurological defects [1]. It is most commonly associated with head trauma, neurosurgical interventions such as ventriculoperitoneal (VP) shunt placement, infections, malignancy, or spontaneous causes [1,2]. Pneumocephalus is classified into two categories: simple pneumocephalus and tension pneumocephalus. Simple pneumocephalus is often asymptomatic, following a procedure such as a craniotomy or burr hole placement. They are benign and self-limiting [3,4]. Tension pneumocephalus occurs when intracranial air accumulates under pressure, causing brain compression and mass effect [4,5]. This leads to increased intracranial pressure (ICP) with symptoms such as headache, altered mental status, nausea, vomiting, neurological deficits, and, in severe cases, death [5]. In patients with VP shunts, pneumocephalus can arise due to altered cerebrospinal fluid (CSF) flow or skull base defects, which create a vacuum for air trapping [6,7]. Limited data exist on the prevalence of this complication; however, prior studies have documented cases in patients with longstanding VP shunts, often presenting with symptoms of shunt dysfunction and elevated ICP [6,8].

Here, we report a case of severe tension pneumocephalus in an elderly patient with a history of VP shunt placement, leading to rapid neurological deterioration and necessitating emergent neurosurgical intervention. This case highlights the unique interplay between VP shunts and cranial defects, demonstrating how shunt-induced negative pressure can exacerbate air entry and accelerate neurological decline, an uncommon but significant teaching point for medical professionals.

## Case presentation

An 83-year-old male with a past medical history of hypertension, hyperlipidemia, diabetes mellitus, depression, normal pressure hydrocephalus status post right VP shunt placed five years ago, and left subdural hematoma (SDH) evacuated one month prior, presented to the Emergency Department via Emergency Medical Services (EMS) as a stroke alert. The family reported he was at baseline when he suddenly became unable to move, exhibiting a blank stare, global aphasia, and inability to follow commands, prompting an Emergency Services (911) call. Baseline function included ambulation with a walker and self-feeding, with assistance needed for other activities of daily living. The family reported no recent trauma, fever, chest pain, or shortness of breath (SOB).

On arrival, he was hemodynamically unstable (BP 167/101 mmHg, HR 53-68 bpm) and on BiPAP with mild respiratory alkalosis (pH 7.43, pCO2 36, pO2 112). The exam revealed disorientation, minimal responsiveness, partial gaze palsy, complete facial paralysis, global aphasia, 0/5 strength, severe sensory loss in all extremities, and an NIHSS Stroke Scale (NIHSS) score of 29. He was normocephalic, atraumatic, with a well-healed left parietal craniotomy scar and no signs of infection or trauma. A chronic heart murmur was noted, with chronicity based on notes from previous hospital admissions. The patient was breathing comfortably on BiPAP, the abdomen was soft, non-distended, and non-tender.

**Figure 1:**
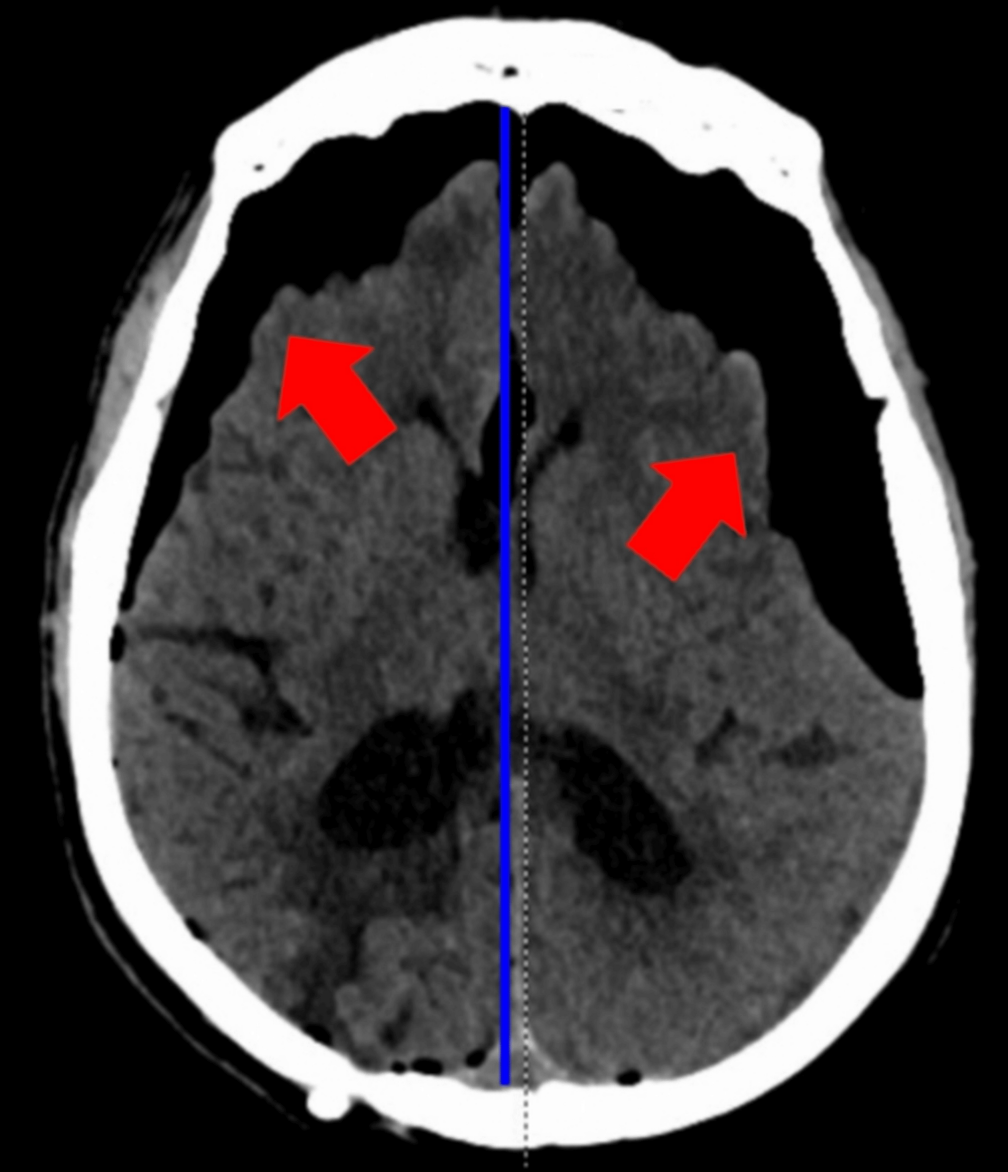
Non-contrast CT brain before neurosurgical intervention, depicting severe frontal pneumocephalus (left greater than right), with air extension in the skull and right parietal encephalomalacia, indicated by the red arrows. There is also a 4 mm rightward midline shift, with the midline depicted by the dashed line and the shifted midline depicted by the blue line.

**Figure 2:**
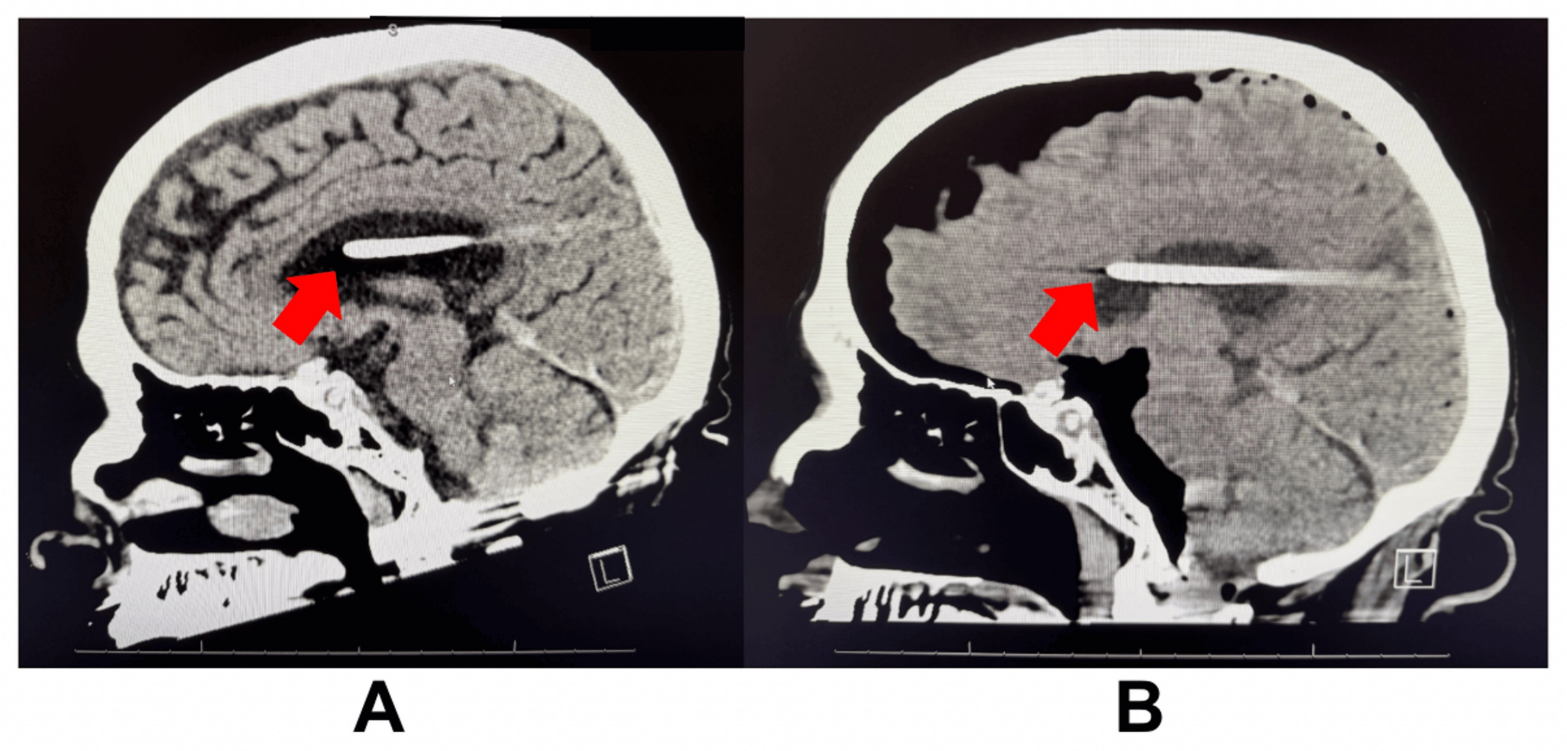
Non-contrast CT brain depicting the shift in VP shunt. Picture A shows the VP shunt inside the lateral ventricle from imaging during a prior admission. Picture B shows the VP shunt shifted slightly anterior compared to the position in picture A. The anterior tip of the VP shunts is indicated by the arrows.

Emergent non-contrast computed tomography (CT) of the brain showed severe frontal pneumocephalus (L>R) with mass effect, a 4 mm rightward midline shift, and right parietal encephalomalacia (Figure 1). The VP shunt appeared slightly anterior compared to prior imaging (Figure 2). The patient was intubated for airway compromise, started on broad-spectrum antibiotics, and neurosurgery was consulted. A VP shunt imaging series was ordered to assess the function and placement of the device, which proved unremarkable. The pneumocephalus was visualized, and the VP shunt catheter had no evidence of fractures.

The neurosurgical team performed an emergent left-sided burr hole evacuation and VP shunt removal via a right posterior occipital incision. The procedure aimed to relieve ICP by allowing air drainage and reducing mass effect. Although a bilateral procedure was initially considered, intraoperative findings suggested adequate decompression with left-sided intervention alone. Postoperatively, he remained in the intensive care unit on mechanical ventilation.

**Figure 3:**
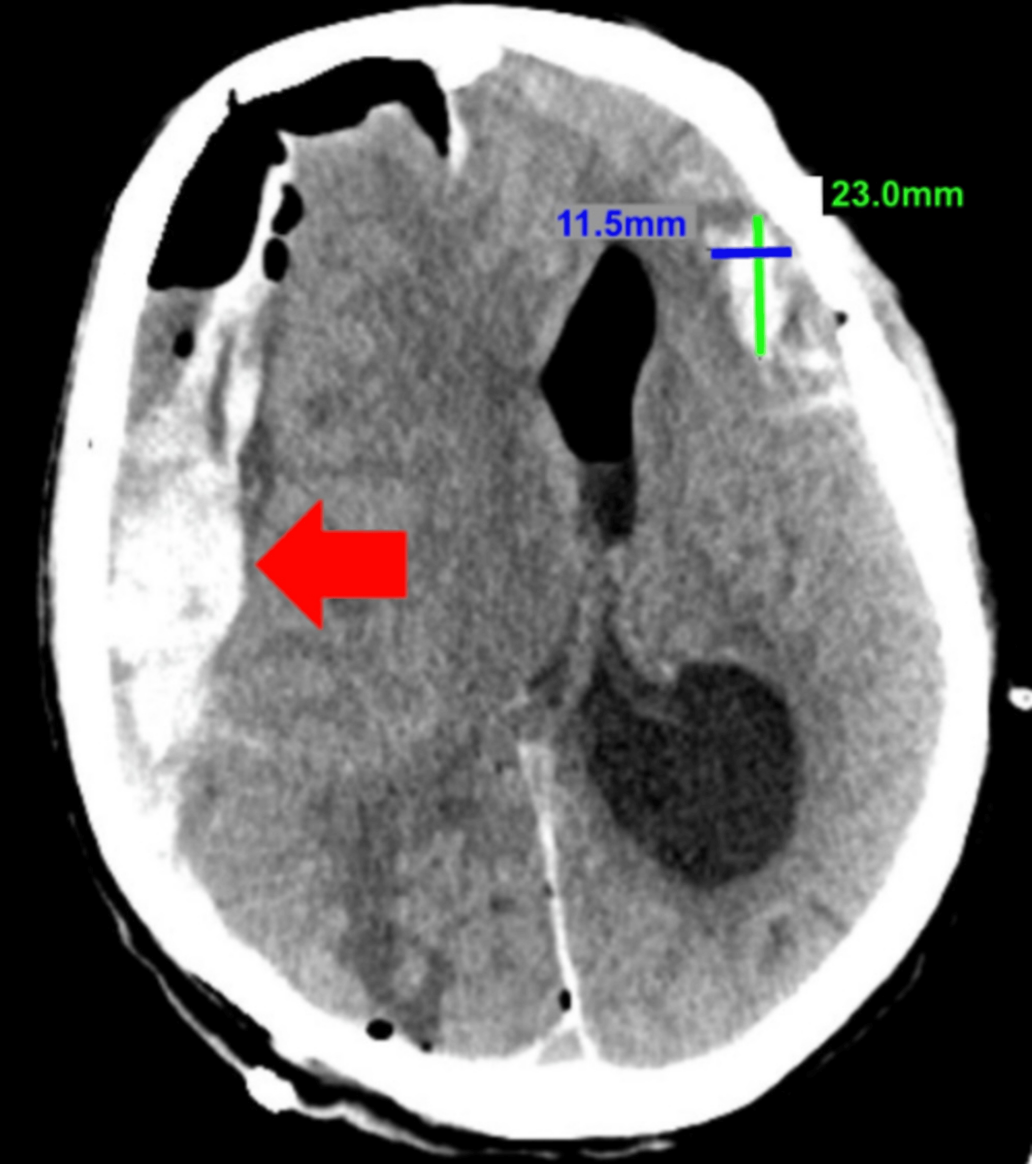
Non-contrast CT brain post-procedure depicting the partial resolution of intracranial air and a new right-sided SDH depicted by the arrow. There is also a small bleed on the left side, measuring 23.0 mm by 11.5 mm. SDH: Subdural hematoma

Despite intervention, his neurological status remained poor. Follow-up CT brain revealed a new right-sided SDH (Figure 3). Neurosurgery discussed the poor prognosis with the patient’s family, who initially opted for maximal medical and surgical management. The patient subsequently underwent a right-sided craniotomy. Post-operatively, he remained unresponsive, with questionable seizure activity. Given the lack of improvement and poor prognosis, the family declined further interventions, and he was transitioned to a do-not-resuscitate status and hospice care, with compassionate extubation performed. Table 1 summarizes the patient’s clinical course from baseline through interventions and outcomes.

**Table 1:** Timeline of Clinical Course, Interventions, and Outcomes in an Elderly Patient with VP Shunt-Associated Tension Pneumocephalus ADL: Activities of daily living; VP: Ventriculoperitoneal; SDH: Subdural hematoma; DNR: do-not-resuscitate

Stages of Patient Presentation	Clinical Status	Imaging/Findings	Interventions	Patient Outcome
Baseline	Alert, oriented, ambulatory with walker; required ADL assistance	Prior right VP shunt, left SDH evacuated 1 month prior	Routine care	Stable at baseline
Acute Presentation	Sudden inability to move, global aphasia, NIHSS 29	Severe frontal pneumocephalus (L>R), 4 mm midline shift, right parietal encephalomalacia; VP shunt slightly anterior	Stroke alert called, BiPAP support	Rapid neurological deterioration
During Hospital Stay	Disoriented and eventually unresponsive	Same as in acute presentation	Left burr hole evacuation, VP shunt removal, intubation, antibiotics, ICU care	Partial resolution of pneumocephalus; continued poor neurological status
Final Outcome	Unresponsive, minimal improvement	Follow-up CT showed partial resolution of pneumocephalus and a new SDH	Right-sided craniotomy for new SDH; later hospice care, DNR	Compassionate extubation; patient deceased

## Discussion

This case highlights the potential development of tension pneumocephalus secondary to micro-openings at a healing burr hole site. This led to air trapping that was rapidly exacerbated by the VP shunt, resulting in acute clinical deterioration. Atraumatic pneumocephalus is a rare and life-threatening complication that warrants prompt recognition and intervention. This case is particularly unique due to its mechanism and rapid progression, offering valuable insight into an uncommon but serious postoperative risk that should be considered in patients with VP shunts and recent cranial procedures.

A month prior to this admission, the VP shunt was placed at a high setting performance of 2.5 (140 mm Hg drainage pressure) to aid the evacuation of the left SDH. A higher shunt pressure setting was chosen to minimize CSF drainage and prevent overdrainage, which can lead to negative pressure in the subarachnoid space and subsequent expansion of the SDH due to tearing of bridging veins or brain shifting. Despite these precautions, the patient required surgical evacuation of the hematoma through a left burr hole. On follow-up visits, a well-healed surgical scar on the left parietal scalp was noted; no skin breakdown or signs of infection. The patient was progressively improving and returned to his domiciliary environment.

Tension pneumocephalus is a known complication of cranial trauma and acute VP shunt placement [2]. However, this patient had no recent head trauma, and the VP shunt was placed over five years ago. This prompted the question of how an acute and atraumatic tension pneumocephalus could develop with such rapid onset and neurological deterioration.

The literature supports that micro-openings in the previous left parietal burr hole surgical scar allowed small amounts of air to enter the intracranial space. The negative pressure from the VP shunt created a vacuum, which facilitated the rapid introduction of air into the brain cavity. This led to the quick expansion and entrapment of air, hence a tension pneumocephalus [9]. The literature also indicates that the combination of a VP shunt and a cranial defect or fistula may provide a route for air to enter the cranial cavity [9,10]. We believe the negative pressure from the VP shunt caused the acute presentation of this tension pneumocephalus and rapid neurological deterioration in the patient. Table 2 below summarizes seven case studies to highlight the similarities and differences between cases of atraumatic pneumocephalus in patients with VP shunts.

**Table 2:** Reported Case Studies and Mechanisms of Atraumatic Pneumocephalus in Patients with VP shunts This table compares published cases of pneumocephalus associated with VP shunts, outlining the proposed mechanisms, timing in relation to shunt placement, anatomical sites of air entry, interventions performed, and key distinguishing features. The cases highlight the similarities and differences between these cases and the importance of vigilance in shunted patients who present with new neurological decline [6,7,11-15].

Article	Mechanism of Pneumocephalus	Onset Timing Post-Shunt Placement	Location of Defect	Interventions	Key Notes/Outcome
Kim et al. (2009) - Otogenic Pneumocephalus Associated with a VP Shunt	Otogenic mechanism: Negative intracranial pressure via VP shunt + mastoid/skull base bony defect	29-30 months post-shunt placement	Posterior fossa, mastoid	Mastoidectomy, Defect repair with muscle plug and bone dust	Patient had tinnitus; successful resolution
da Silva et al. (2021) - Intraventricular Pneumocephalus as a Complication of VP Shunt	Combination of shunt siphon effect + anatomical defect enabling air entry	Greater than 10 years post-shunt placement	Bony defect and meningocele (fistula) at the base of the skull	Bicoronal craniotomy; meningocele closure; frontal sinus cranialization; new VP shunt placed	Specifically discusses air entering the skull when nasal pressure is greater than intracranial pressure when the basal structures are connected to the paranasal sinuses via an opening
Pieri et al. (2011) - Delayed Otogenic Tension Pneumocephalus Complicating VP Shunt	Air entry via petrous apex/tegmen tympani defect + shunt effect	12 months post-shunt placement	Petrous bone/temporal region	Temporarily raised shunt pressure; surgical defect repair	Emphasizes surgical correction of anatomical defect, complicated by aneurysmal subarachnoid hemorrhage
Monas et al. (2010) - Spontaneous Tension Pneumocephalus Resulting From a Scalp Fistula in a Patient With a Remotely Placed VP Shunt	Shunt negative pressure + skull defect (scalp fistula)	4-5 years post-shunt	Frontal parietal region	Percutaneous decompression in the ED, scalp wound debridement and skin closure, broad-spectrum antibiotics	Skull defect due to infection eroding through the overlying tissue of an old burr hole
Verhaeghe et al. (2018) - Delayed Intraventricular Pneumocephalus Following Shunting for Normal-Pressure Hydrocephalus	Bone erosion + shunt negative pressure	10 months post-shunt	Left temporal region/mastoid	Tegmen repair with flap	Delayed complication due to bone erosion secondary to long-standing intracranial pressure
Gkasdaris et al. (2024) - Spontaneous Intraventricular Tension Pneumocephalus	Chronic intracranial hypertension + thinness of bony area at the superior edge of the petrous pyramid	Multiple VP shunt placements; time frame not listed	Between left temporal bone and tegmen tympani; intraventricular	Middle cranial fossa surgery with repair of osteo-meningeal breach	Past medical history of tumor in the pineal region
Alalawi et al. (2025) - Atypical Presentation of Pneumocephalus Post-VP Shunt in a Patient with a History of Endoscopic Endonasal Skull Base Approach: A Case Report	Siphon effect from VP shunt/excessive negative intracranial pressure + postoperative skull base defect	3 days post-shunt	Right petrous apex/middle cranial fossa with brainstem compression	Endoscopic skull base defect repair, fat graft, excessive irrigation	Large extra-axial tumor in the right middle cranial fossa → tumor removal → acute hydrocephalus due to meningitis → VP shunt → pneumocephalus

The patient's presentation and subsequent imaging findings highlight the importance of careful monitoring and timely intervention in patients with VP shunts who are experiencing neurological deterioration. Adjusting the shunt pressure settings to prevent overdrainage and regular follow-up imaging can help mitigate the risk of complications such as SDH expansion and pneumocephalus. This case underscores the need for a multidisciplinary approach, involving neurosurgery, critical care, and infectious disease specialists, to optimize patient outcomes in complex cases involving VP shunts.

## Conclusions

Tension pneumocephalus is a rare but serious complication. Patients with VP shunts who underwent additional cranial procedures appeared to be at higher risk due to potential micro-openings. This case illustrates how a healing cranial defect, combined with shunt-induced negative pressure, can rapidly lead to life-threatening neurological decline. Early recognition and timely neurosurgical intervention are crucial. This case reinforces the need for vigilance in patients with VP shunts who develop new neurological decline, emphasizing the importance of considering pneumocephalus in the differential diagnosis even in the absence of trauma.
